# Global multi-layer network of human mobility

**DOI:** 10.1080/13658816.2017.1301455

**Published:** 2017-03-13

**Authors:** Alexander Belyi, Iva Bojic, Stanislav Sobolevsky, Izabela Sitko, Bartosz Hawelka, Lada Rudikova, Alexander Kurbatski, Carlo Ratti

**Affiliations:** ^a^ SENSEable City Laboratory, SMART Centre, Singapore, Singapore; ^b^ Faculty of Applied Mathematics and Computer Science, Belarusian State University, Minsk, Belarus; ^c^ SENSEable City Laboratory, Massachusetts Institute of Technology, Cambridge, MA, USA; ^d^ Center for Urban Science + Progress, New York University, Brooklyn, NY, USA; ^e^ Department of Geoinformatics – Z_GIS, GISscience Doctoral College, University of Salzburg, Salzburg, Austria; ^f^ Department of Intelligent Software and Computer Systems, Yanka Kupala State University of Grodno, Grodno, Belarus

**Keywords:** Human mobility, Flickr, Twitter, multi-layer network, community detection

## Abstract

Recent availability of geo-localized data capturing individual human activity together with the statistical data on international migration opened up unprecedented opportunities for a study on global mobility. In this paper, we consider it from the perspective of a multi-layer complex network, built using a combination of three datasets: Twitter, Flickr and official migration data. Those datasets provide different, but equally important insights on the global mobility – while the first two highlight short-term visits of people from one country to another, the last one – migration – shows the long-term mobility perspective, when people relocate for good. The main purpose of the paper is to emphasize importance of this multi-layer approach capturing both aspects of human mobility at the same time. On the one hand, we show that although the general properties of different layers of the global mobility network are similar, there are important quantitative differences among them. On the other hand, we demonstrate that consideration of mobility from a multi-layer perspective can reveal important global spatial patterns in a way more consistent with those observed in other available relevant sources of international connections, in comparison to the spatial structure inferred from each network layer taken separately.

## 1. Introduction

People travel from one country to another for different reasons and while doing so, a lot of them leave their digital traces in various kinds of digital services. This opens tremendous research opportunities through the corresponding datasets, many of which have already been utilized for different research purposes, including mobile phone records (Calabrese and Ratti 2006, Ratti *et al*. 2006, Girardin *et al*. 2008, Quercia *et al*. 2010), vehicle Global Positioning System (GPS) traces (Kang *et al*. 2013, Santi *et al*. 2014), smart cards usage (Bagchi and White 2005, Lathia *et al*. 2012), social media posts (Java *et al*. 2007, Frank *et al*. 2013, Szell *et al*. 2014) and bank card transactions (Sobolevsky *et al*. 2014b, 2014c, 2015b, 2016). By looking at these traces, we can reconstruct people’s movements and afterwards analyze them to see if interesting or useful patterns emerge or to build models for predicting where people will travel next.

It has already been shown that results of such analysis can be applied to a wide range of policy and decision-making challenges, such as regional delineation (Ratti *et al*. 2010, Sobolevsky *et al*. 2013) or land-use classification (Pei *et al*. 2014, Grauwin 2015a). A number of studies focus specifically on looking at human mobility at urban (González *et al*. 2008, Hoteit *et al*. 2014, Kung *et al*. 2014), country (Amini *et al*. 2014) or global scale. When considering aspects of human mobility at global scale in particular, two major types of movements can be observed: an international migration (Greenwood 1985, Fagiolo and Mastrorillo 2013, Abel and Sander 2014, Tranos *et al*. 2015) and short-term trips explored for example through geo-localized data from Twitter (Hawelka *et al*. 2014, Sobolevsky *et al*. 2015a) or Flickr (Paldino *et al*. 2015, 2016, Bojic *et al*. 2015b, 2016).

Some studies tried primarily to explain and model global mobility (Greenwood 1985, Fagiolo and Mastrorillo 2013, Abel and Sander 2014, Tranos *et al*. 2015), while others rather focused on its applications, such as revealing the structure of the global society through the global mobility networks (Sobolevsky *et al*. 2013, Hawelka *et al*. 2014). Some scholars even considered relationships between human migration and economic links between countries (Fagiolo and Mastrorillo 2014, Sgrignoli *et al*. 2015). However, global human dynamics shows a complex nature containing various types of mobility, including such different processes as permanent relocations and short-term visits, and thus, cannot be fully understood through any single data source focusing on just one particular aspect of human behavior. Moreover, different sources might reveal different aspects of mobility also possessing different biases and limitations, so one source might complement another. In addition, recent studies provided methodological background to deal with multi-layer complex networks (Kivelä *et al*. 2014) which allows us to utilize their findings to build one network by combining different data sources.

In this study, we use three different sets of data each representing different kind of people’s movements. Namely, Flickr and Twitter represent short-term human mobility (Sobolevsky *et al*. 2015a), while migration network contains information about a long-term one. Although Flickr and Twitter are similar in a certain sense, they show different types of people’s activity, their usage while traveling and while at home also differs and they might correspond to movements motivated by different reasons (Li *et al*. 2013). Namely, Flickr mostly reflects activity during a leisure traveling and sightseeing, while in case of Twitter, its data mostly reflect activity during spare time with internet access available which can be performed during business trips as well as leisure traveling (Kiss 2011). Moreover, they are complement, as in some countries only one of these services may be popular and widely used by people.

Further, we provide a comparative study of different layers of human mobility. The specific focus of our study is on demonstrating that such a complex approach to human mobility, considering it from different short- and long-term perspectives, is of a vital importance: a multi-layer global mobility network shows patterns not seen from each layer separately. In order to evaluate our hypothesis, we applied a method of detecting communities in multi-layer networks and compared outcomes with those for the other existing international connections (language similarities, present or former colonial relations as well as trade networks). The results showed that communities detected in the three-layer network are on average much more similar to communities in the language, colony and trade networks than the ones observed in each layer separately.

## 2. Datasets

As our study aims at investigating human mobility from two different perspectives (i.e. long-term and short-term), we include three datasets where two of them capture short-term human movements such as touristic, personal or business travel, and one of them reflects long-term mobility such as people moving to another country to live there. In that sense, short-term human mobility is inferred from more than 130 million geo-tagged digital objects (e.g. videos and photographs) publicly shared on Flickr and more than 900 million geo-tagged tweets posted by 13 million users on Twitter, while long-term one is revealed from the United Nations official migration statistics (further discussion on the nature of human mobility represented by these datasets could be found in the Supplementary Information). Moreover, in order to compare mobility patterns determined in the aforementioned way and in some sense also to verify our results, we used three other datasets showing international connections: colonial dependency network, a network of languages shared by countries and a network of international trade.

The Flickr dataset used in our study contains more than 130 million photographs/videos and was created by merging two publicly available Flickr datasets – one coming from a research project (Mount 2010) and another from Yahoo^1^ (Thomee *et al*. 2016). The records in two datasets partially overlap, but since each digital object in both datasets has its id, we were able to merge them by omitting duplicates and choosing only those records that were made within a 10-year time window, i.e. from 2005 to 2014. Since the Flickr data are quite sparse, we use 10 years’ time span to gather more data and to avoid possible biases that could be caused by the dataset’s sparseness. In Section 6 of Supplementary Information, we provide more in-depth discussion about the choice of the time frame and show that this choice does not affect results of our analysis. The second dataset on short-term mobility consists of the geo-tagged messages posted during 2012 and collected from the digital microblogging and social media platform Twitter. The data were collected with the Twitter Streaming API (Twitter 2013) and cleansed from potential errors and artificial tweeting noise as previously described by Hawelka *et al*. (2014).

In order to build a two-layer directed and weighted network that describes short-term human mobility, we had to convert the Flickr and Twitter datasets into origin–destination matrix where origins represent users’ home countries and destinations are the places (i.e. countries) where users created digital objects or tweeted from. Since both datasets do not contain information about user home location, we had to determine it. Results from previous research showed that it is important to use the proper method for home location definition based on the context of the data (Bojic *et al*. 2015a). Taking this into account, we chose the most conservative method from techniques used in similar studies. Namely, we decided who of the users are acting in each location as residents based on the following criteria: a person is considered to be a resident of a certain country if this is the country where he/she took the highest number of the photographs/videos over the longest timespan (calculated as the time between the first and the last photograph taken within the country) compared to all the other countries for this person. Moreover, we omitted all users for whom timespan was shorter than 180 days (i.e. roughly 6 months) and who took less than 10 photographs/made less than 10 tweets. Usage of the timespan of at least 6 months ensures a high probability of preventing most of the cases of wrongly detected home location for Flickr users who just happened to demonstrate high activity during their occasional travel to a certain destination (which happens often for tourists getting excited about the place they visit), as we require them to perform repeated activity in their prospective home country over an extensive period of time. However, even though our method is more conservative compared to other methods used in the literature, it can still produce errors. We believe, however, that amount of such errors is low enough so that it does not affect the validity of the aggregated country-to-country mobility analysis.

Using this simple criterion, we were able to determine the home country for over 500 thousand users in the Flickr dataset who took almost 80% of all the photographs/videos in the dataset (i.e. more than 90 millions in total), while the rest of the users, for whom home country could not be defined, mostly belong to a low-activity group taking photographs only occasionally. As described by Hawelka *et al*. (2014), all Twitter users were considered as residents of the country where they were most active. When constructing our multi-layer weighted and directed mobility network, we only considered users for whom we were able to determine their home country. Finally, two countries are connected with a link if there is at least one person from the first country that had some activity in the second country where the value of every weighted link in this network corresponds to the total number of users from one country that made digital objects or tweeted in the other one.

**Figure 1.**
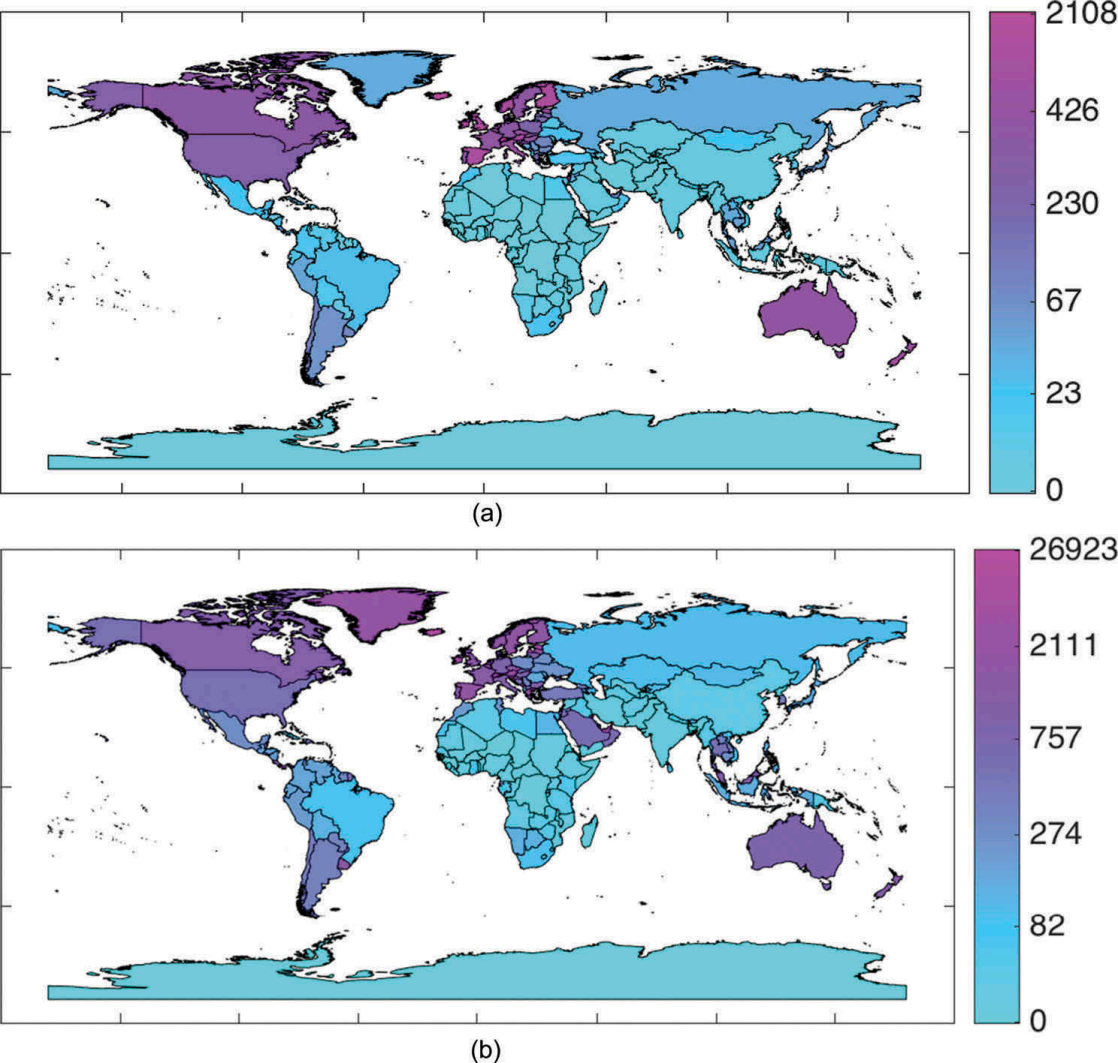
Penetration of (a) Flickr and (b) Twitter into countries all over the world as number of users who travel abroad per one million of population.

We should mention here that Flickr and Twitter are much more widely used in developed countries while penetration into some other countries can be quite low. Figure 1 shows how many users per one million of population from each country we determined to be active outside their homeland. We can see that penetration in China (mostly due to restrictive legal regulations) and India as well as in the most African countries is pretty low. About 75% of all the countries for Flickr and 45% for Twitter have less than 0.01% of their population ever using these resources abroad. However, this penetration rate of travelers recorded in the datasets can go up to couple of percent for some countries, while on average about 5% of the Twitter users and 3% of the Flickr users, captured by geo-located content they posted on corresponding service, were recorded traveling abroad.

The third layer was constructed using a dataset with statistics on number of foreign citizens or foreign-born population living in each country in July 2010. These data are publicly available and can be downloaded from the United Nations Department of Economic and Social Affairs website (United Nations 2015). This statistic is basically already provided in a form of an origin–destination matrix, making the process of extending the two-layer directed and weighted network on human mobility with one additional layer describing long-term human movements a very straightforward one.

Finally, to see how the constructed three-layer mobility network correlates with cultural and economic parameters, we created three separate networks of country relationships based on colonial dependence, common language spoken by people in different countries and bilateral trade between countries. In the first network of colonial dependence (Hensel 2009), two countries are connected if one of them was a colony or dependent territory of another one. Furthermore, the second network is a network of common spoken languages (Infoplease 2015) where two countries are connected if there is at least one common language that is official in both countries or spoken by the majority of the population in both of them. Unlike the first two, the third one is a weighted network of trade flows between countries obtained from the United Nations Commodity Trade Database (United Nations 2014) where the value of the link represents the amount of import/export (in US dollars) in 2012.

## 3. Quantitative and qualitative properties of mobility networks

Before getting to the analysis of the entire three-layer mobility network, constructed in the way explained in the previous section, we start from a comparative study of some basic quantitative characteristics of the network layers. We look at their differences and similarities, investigating if those different datasets effectively tell us the same stories about global human mobility or the different ones. Specifically, the main focus of this section is on the comparison of the long-term versus short-term attractiveness of the countries. Focusing on the countries’ ability to attract foreign visitors, for this analysis we excluded loop edges from all the networks and considered a common measure of ‘incoming degree centrality’, which in our case of weighted networks becomes ‘strength centrality’. We additionally consider the distribution of links’ weights in order to gain insights from the overall composition of the international mobility fluxes.

**Figure 2.**
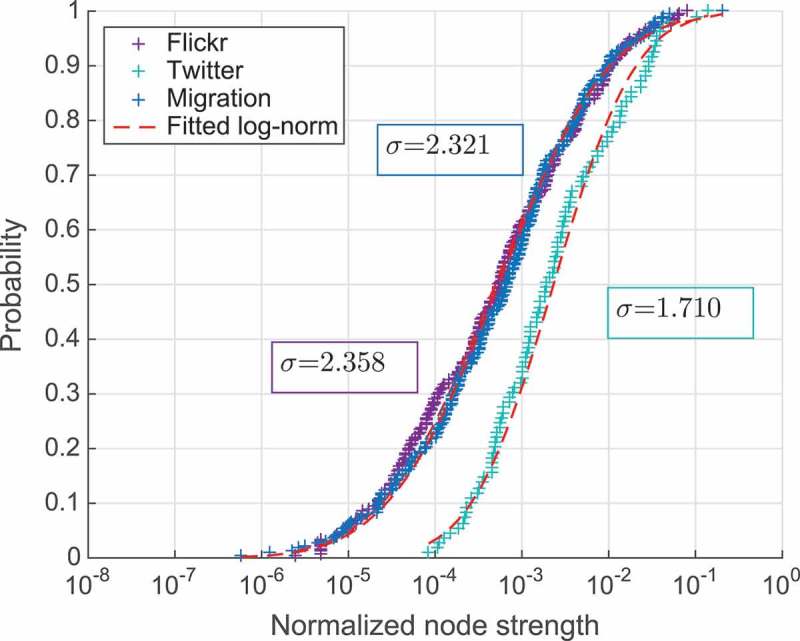
Cumulative distribution of normalized nodes’ strengths.

**Figure 3.**
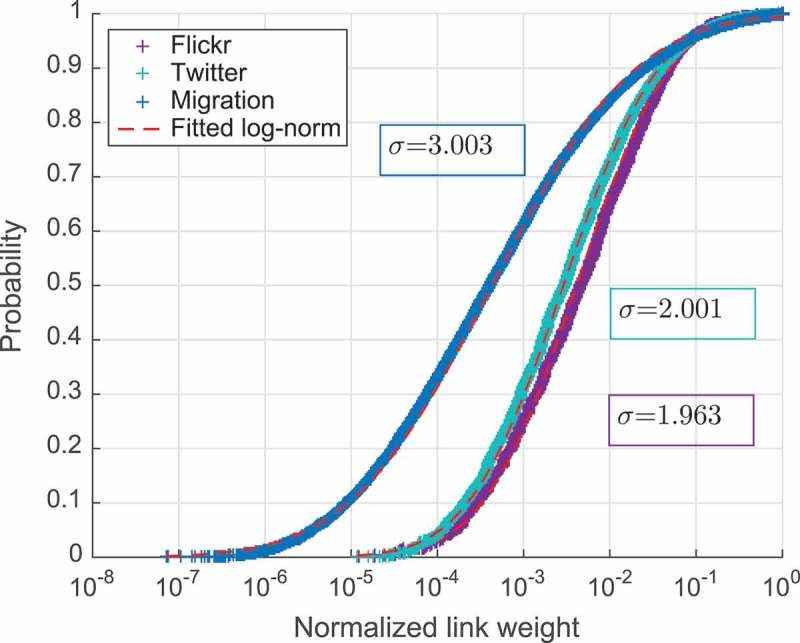
Cumulative distribution of normalized links’ weights.

Relative country attractiveness for the foreigners can be defined as the fraction of all people who travel outside their country of origin and come to that considered country of destination. It can be approximated as a normalized node incoming strength (i.e. as the sum of weights of all incoming non-loop edges to the given destination divided by the sum of all non-loop edge weights in the network). Strictly speaking this metric only represents a proxy to the actual country’s attractiveness and is dependent on the representativeness of the data used to construct the corresponding mobility network. In the case of Twitter and Flickr, the last might be largely affected by the heterogeneity of the data coverage across the world. However, we believe that being evaluated at the global scale this metric is still useful for the relative comparison of the attractiveness of different countries, at least in the context of the social media activity. We also consider relative weights for each specific mobility flux between the two countries as the number of people moving between them normalized by the total number of people moving out of the considered origin. Based on Figure 2, which plots cumulative distribution function of normalized incoming strengths of the nodes, and Figure 3, that plots relative link weights for all three networks, we can conclude that all shown distributions are pretty similar to log-normal. Plot in Figure 3 shows that the distribution of links’ normalized weights in the migration network has a value of variance much higher compared to two touristic networks. This means that the migration fluxes from each country are generally more diverse than the short-term mobility ones as seen from Flickr and Twitter networks.

**Figure 4.**
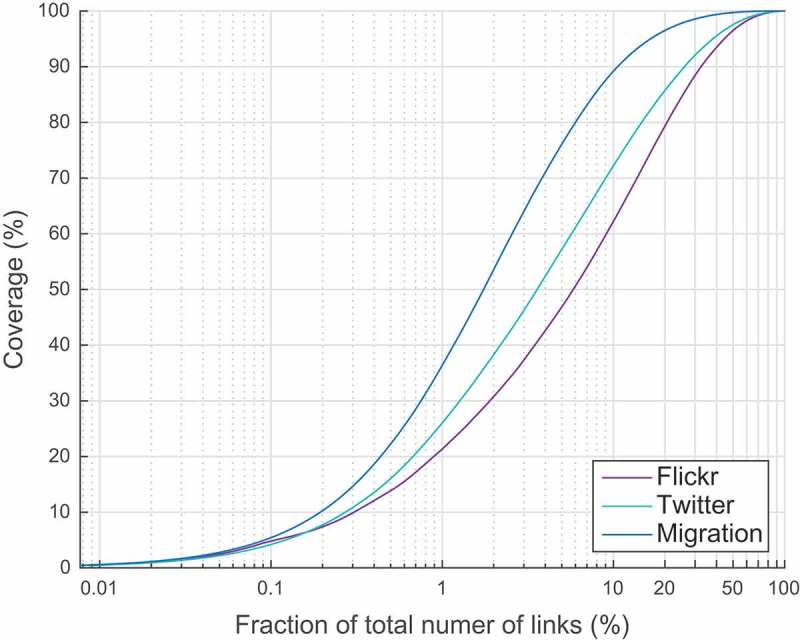
Flow coverage.

In order to explore the observed link diversity in more details, in Figure 4 we show a cumulative sum of the links’ weights sorted in decreasing order. From this plot one can see what percentage of the entire network’s flow is covered by a certain number of the heaviest links. For example, we can see that in the migration network top 1% of the links cover almost 40% of the entire network flow, i.e. total weight of 1% of the heaviest links equals almost 40% of the sum of all weights, and 10% of the links cover 90% of the flow. At the same time, those values are much smaller for the other two networks: 1% of the links cover about 20% and 25% of the flow and 10% cover only about 60% and 70% of the entire flow in the Flickr and Twitter networks, respectively. This observation can be explained by the fact that although locally for each country migration links are more diverse, globally for tourism there is a much broader choice of major destinations than for migration.

**Figure 5.**
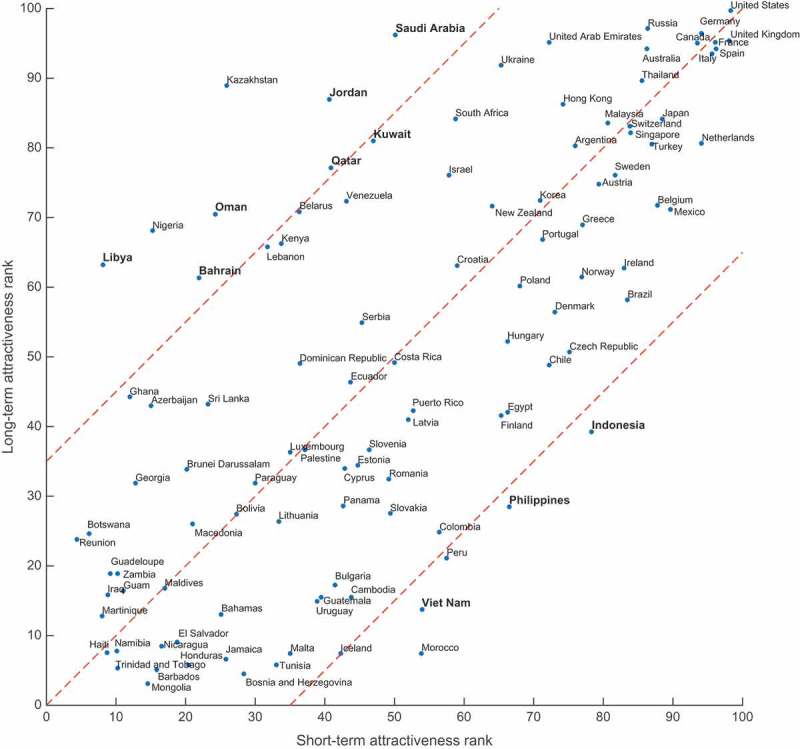
Comparison of countries’ short-term vs. long-term attractiveness ranks.

Finally, we calculate countries’ ranks according to their foreign attractiveness (more details could be found in Supplementary Information). Figure 5 shows how a short-term attractiveness rank (averaged over Twitter and Flickr networks, to diminish heterogeneity of their coverage) correlates with a migration attractiveness rank. However, interesting stories are told by the outliers. Here we can see some very clear patterns. On the one side, there are prosperous Middle East countries like Saudi Arabia, Qatar, Oman, Bahrain, etc., that attracted huge amount of foreign manpower during the last century after vast reserves of oil were discovered in the region. In many of these countries, number of immigrants exceeds the local population. While being that attractive for professional migration, they are relatively less popular for short-term visitors, including tourists. And on the other side, there are less prosperous countries like highly populated Southeast Asian countries: Indonesia, Philippines and Vietnam, not so much attractive for migrants, but relatively cheap for tourists and offering very interesting natural attractions such as Bali and Phu Quoc, just to name a few, which makes them primary touristic destinations. Needlessness to say, there are also highly developed countries such as the United States, the United Kingdom, Germany, etc., with very well-established tourism, plenty of business visitors and lots of incoming migrants, being highly ranked in both short- and long-term attractiveness. In Supplementary Information, we also compare countries by their per capita performance in attracting foreign visitors measured as a number of visitors divided by country’s population. This metric might be useful for the analysis of the relative strengths of personal and business ties, which are strongly related to the number of people living in the country.

Although results of our analysis presented in this section showed that all three networks share some common properties, at the same time they differ in many aspects. Common features of networks reflect certain facts about different types of countries: the most developed countries are popular for both types of visitors, but then again there are also countries attractive only from one side. Moreover, it has been shown that the migration flow is much more concentrated around just couple of pairs of origin/destination countries, while tourists have a larger variety of choices when deciding which place to visit next.

## 4. Modeling mobility

One common goal that many studies on human mobility share is to predict mobility flows. Although over time several different models were proposed, still most of the related works rely on the classical gravity model (Zipf 1946, Barthélemy 2011). The model takes spatial population distribution including distances between different locations as an input and predicts mobility fluxes with respect to several model’s parameters. The values of those parameters are either assumed or have to be estimated from partial knowledge of the network. Recently, an alternative parameter-free radiation model has been suggested (Simini *et al*. 2012), which allows to predict human mobility just based on the spatial distribution of the country population without any parameters to fit. However, the radiation model in turn relies on some partial knowledge of the mobility network as specified below. We compare performance of these models on our global mobility network and use them to reveal and compare patterns behind the three layers of this multi-layer network.

Classical gravity model tries to predict number of people moving from the origin *i* to the destination *j* as 
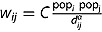
, where 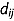 is the distance between *i* and *j*, 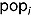 is the population of *i, C* is a global normalization constant ensuring that the predicted total activity is the same (or on the same scale) as observed and 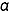 is an adjustable parameter of the model. However, while the populations of the destinations are useful for understanding how the outgoing mobility from each origin should be distributed, the population of the origin might not be the most relevant parameter for our networks due to high differences in penetration level of Flickr and Twitter across populations of different countries. To address this network heterogeneity, we used the total amount of outgoing mobility 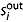 observed in the network instead of the population 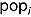 of the origin *i*. This will also help for a fair comparison between gravity and radiation models because the last, as we will describe below, specifically relies on the knowledge of 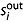. Therefore, in our case the final expression for predicting a flux from *i* to *j* is given by

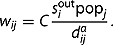

We also consider a ‘locally normalized’ version of the gravity model, i.e. gravity model in a form:

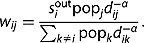

This type of constrained model is rooted in the earlier work of Wilson (1967) and more recently presented by Sagarra *et al*. (2013) and Grauwin *et al*. (2015b).

Unlike gravity, radiation model is claimed to be parameter-free. It uses only population distribution to predict the flux of people as

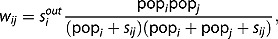

where 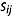 is the population within circle with center at *i* and radius equal to the distance between *i* and *j* excluding population of *i* and *j*, and 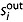 as before represents the total number of the commuters from *i*. However, it is worth mentioning here that the model still depends on the knowledge of 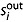. Masucci *et al*. (2013) adjusted the radiation model, introducing the appropriate normalization factor for finite systems. After incorporating this factor, we came up with equation that we used in our experiments in a form of

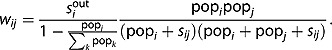

**Figure 6.**
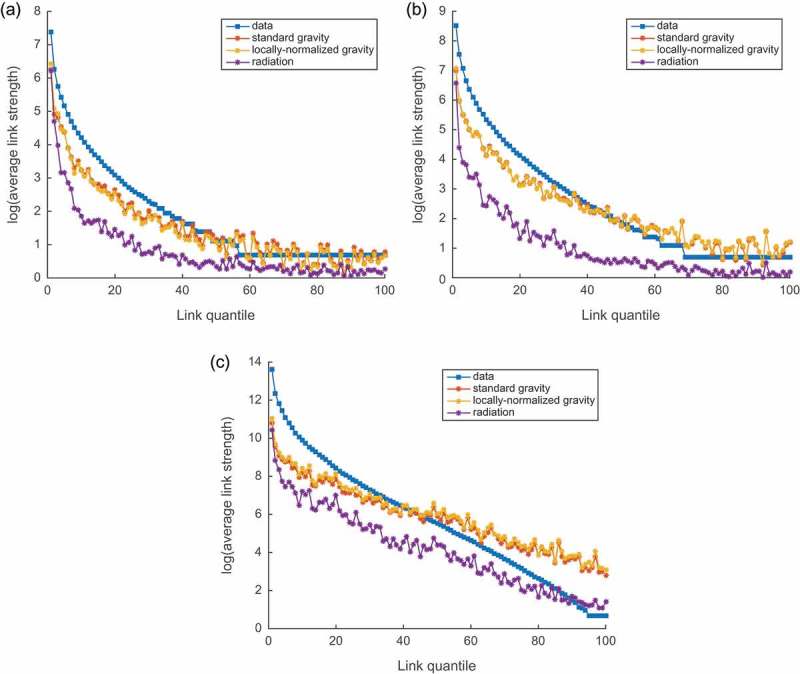
Fit of the models to (a) Flickr, (b) Twitter and (c) migration networks.

After fitting the gravity model and its ‘locally normalized’ version on the logarithmic scale, together with constructing the radiation model for the three layers of our multi-layer mobility network, we got results presented in Figure 6. From the figure and values of 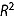 presented in Table 1, we can conclude that for all three layers the gravity model – both classical and locally normalized versions – fit much better for the appropriate choice of the parameters. For that reason, in our further analysis we will only use the gravity model.

**Table 1.** Results of fitting models to layers.

Parameter	Flickr	Twitter	Migration
gravity,	1.253 [1.237, 1.270]	1.112 [1.097, 1.128]	2.009 [1.993, 2.025]
‘locallynormalized’ gravity,	1.431 [1.415, 1.448]	1.160 [1.144, 1.177]	2.178 [2.161, 2.196]
gravity	0.626	0.743	0.774
‘locallynormalized’ gravity	0.649	0.743	0.767
radiation	0.444	0.413	0.649

Worth mentioning is that sometimes other parameters are introduced to the gravity model as well, such as population exponents. However, adding them to the model did not improve performance much, while their values appeared to be pretty close to 1, so we limit our analysis with having just the distance exponent making the highest impact and revealing the strongest pattern.

In Table 1 we report values obtained while fitting parameter 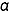, i.e. the exponent for the impact of the distance between origin and destination countries. Those values indicate how fast the flux of people moving between two countries decays with an increment of the distance between them. As one can see from the table, there is a pattern that happens to be pretty distinctive for different layers of the network. And it looks quite consistent for both classical and locally normalized versions of the model: the exponent is slightly higher for Flickr network compared to Twitter and much higher for the migration layer. The higher the exponent is, the faster the decay of mobility with the distance is. We thus can conclude that migrations are usually much more dependent on distance, while short-term mobility is much less spatially constrained.

## 5. Detecting communities in the three-layer mobility network

After exploring properties of each network (layer) separately and discovering certain general similarities as well as some noticeable differences between the layers, we wanted to check our hypothesis that the three-layer mobility network can reveal qualitative patterns that cannot be observed by looking at only layer by layer. In order to evaluate the hypothesis, we focus on such a key pattern, one can discover by means of the mobility networks, as the community structure of the human society. We aim to show that combining a long-term mobility, such as migration, with short-term travels, captured by social media data, into a network with multi-layered structure can reveal important spatial patterns that might not be described by studying any single aspect of the human mobility alone.

Previous studies (Ratti *et al*. 2010, Sobolevsky *et al*. 2013) have shown that community detection in human interaction and mobility networks usually leads to connected spatially cohesive communities (even with no spatial considerations in the community detection method) often revealing meaningful geographical patterns. This was no exception for the global mobility networks estimated from Twitter (Hawelka *et al*. 2014), as well as from migration data (Fagiolo and Mastrorillo 2013). However, while separate layers of mobility network provide interesting partial insights on the spatial structure of the global human society, we wonder if certain patterns can be seen only from the multi-layered perspective. Several ways to detect communities in multi-layer networks were proposed recently (Mucha *et al*. 2010, Tang *et al*. 2012) and for our study we chose the approach based on a direct multi-layer generalization of the most widely used objective function for network partitioning which is modularity (Newman and Girvan 2004, Newman 2006).

Even before building a multi-layer generalization of the modularity, one more adjustment has to be made to it in order to account for the absence of the loop edges in the mobility networks we consider. For that purpose, we altered the way null model used by modularity estimates the weight of each edge. In its classical form, modularity uses 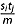 as an expected weight of the edge from an origin node *i* to a destination node *j*, where 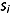 and 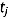 are in- and out-strengths of the nodes *i* and *j*, respectively, and *m* is the total weight of all edges, i.e. 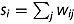, 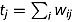 and 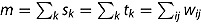, while 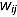 is the observed weight of the link from *i* to *j*. One of the possible explanations of such expected value 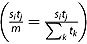 is that in random network that would preserve nodes’ degrees or strengths, distribution of the outgoing strengths 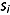 among all the possible destinations is proportional to these destinations’ incoming strengths 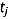. Another one (as it is also 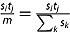) is that distribution of the incoming strength 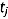 among all the possible origins is proportional to these origins’ outgoing strengths 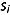. However, if loop edges do not participate in this distribution, then it should be rather 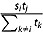 or 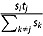, depending on whether it is seen as distribution of the outgoing strength 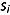 among all the destinations except *i* itself, or as distribution of the incoming strength 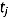 among all the origins except *j*. Finally, as an ultimate estimation we use average of those, that leads to the expression 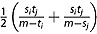.

Since it has already been shown that modularity suffers from certain drawbacks, such as a resolution limit (Fortunato and Barthélémy 2007, Good *et al*. 2010) preventing it from recognizing smaller communities, we also used the approach proposed by Arenas *et al*. (2008) that involves introduction of the so-called resolution parameter, leading to further adjustment of the modularity score. This way the final formula for the adjusted modularity measure used for our case of the mobility networks free of the loop edges is:

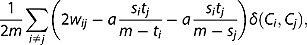

where *a* denotes the resolution parameter, *i, j* are nodes, 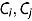 – the communities they belong to, 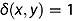 if 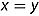, 0 otherwise.

To deal with the multi-layer network, where all layers share the same nodes, we followed the approach proposed by Tang *et al*. (2012). We combined adjusted modularity scores of each layer, taking their average value, and used this as a resulting utility function for the multi-layer network as follows:

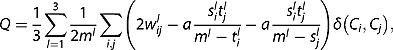

where *l* denotes layer, 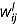 is the weight of the link from *i* to *j* in layer *l*, 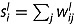, 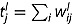, 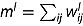.

Worth mentioning is that being defined as a normalized metric, modularity allows to focus on the qualitative structural properties of the network, largely addressing potential heterogeneity of the coverage of the data used to represent human mobility. This is done by evaluating each network edge versus the null model expectation, taking into account actual activity levels of the origins and destinations.

**Figure 7.**
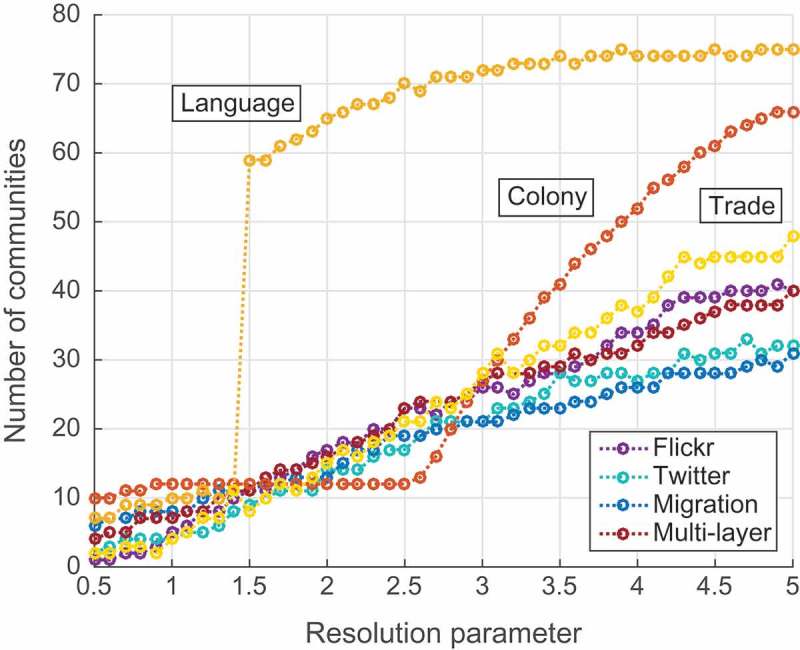
Number of communities depending on resolution parameter.

In order to find the best partition, we optimized this multi-layered version of modularity using efficient and precise Combo algorithm (Sobolevsky *et al*. 2014a), suitable for dealing with different types of objective functions. For the sake of noise reduction, we excluded nodes for which incoming or outgoing strength was less than 10 at least in one layer, that left us with a network of 201 countries and territories. We consider partition of each of the three network layers separately and of the entire three-layer mobility network with different values of resolution parameter. Figure 7 shows dependence of the resulting number of communities (provided by the algorithm looking for the optimal partition in terms of the adjusted modularity for any number of communities) on a value of the resolution parameter. As it can be seen from the figure, for the range of values of resolution parameter between 0.5 and 5.0, the number of obtained communities varies from 1, meaning that the whole network is represented as one community, to more than 60–70 for some of the networks, when most of communities consist of 1–3 countries and further analysis becomes pointless (in the Supplementary Information we present extended plots for resolution parameter values in the range from 0.5 to 10.0 showing that further outcomes are not affected in principle by broadening the range).

In order to provide the evidence for our hypothesis, we wanted to quantitatively evaluate obtained partitions. For that purpose, we compare them with the partitions obtained from other types of international connections. We quantify the similarity between partitions using the normalized mutual information (NMI) (Ana and Jain 2003, Danon *et al*. 2005), a similarity measure which came from information theory and now is widely used in community detection for comparison of partitions. After expansion of all expressions for entropy in its definition, NMI of two partitions *A* and *B* can be calculated as:

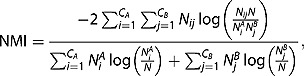

where 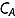 and 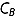 are the numbers of communities in each partition, 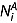 and 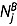 are the cardinalities of each community, 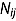 are the numbers of nodes classified to community *i* in partition *A* and to community *j* in partition *B* and *N* denotes the total number of nodes. NMI takes values from 0 to 1 and the higher its value is, the more similar the partitions are, meaning that for identical partitions NMI equals 1.

**Figure 8.**
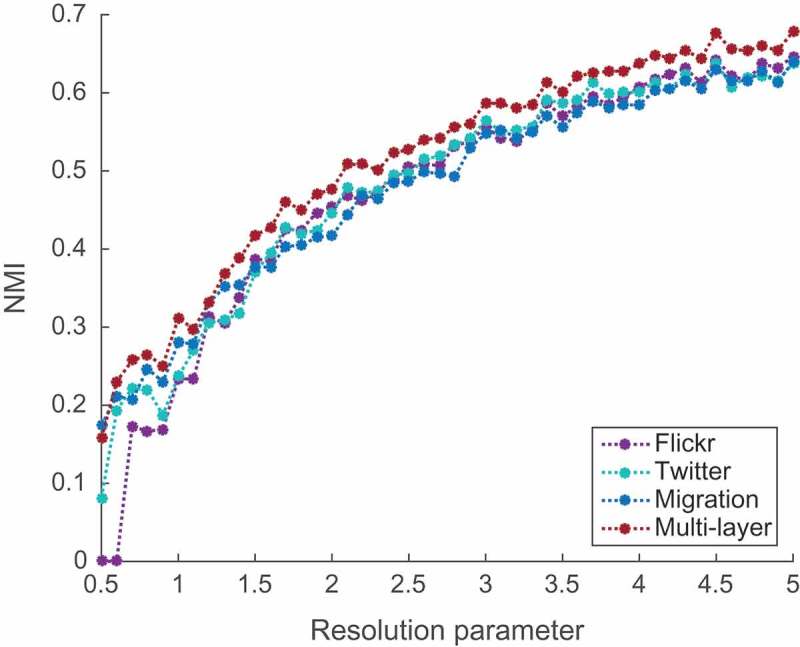
Similarity of community structure between networks of human mobility and other existing international connections.

As it was previously described in Section 2, we chose language, colonial and trade networks as representatives of non-mobility types of international connections. We should mention though that data for constructing the language and colony networks were obtained from the results of other studies and might not represent an ultimate ground truth. Since the nature of these networks is quite different compared to the mobility networks, a direct quantitative link-by-link comparison would not make sense. Instead, we focused on the comparable patterns one can actually discover from different types of networks, such as community structure. We compared partitions of each layer and the entire multi-layer network with partitions of three other networks for the corresponding values of the resolutions parameter, controlling the overall scale of the partition in each case. We quantified similarity of partitions and took average NMI to evaluate how consistent the partitions of each layer and the entire multi-layer network are with the patterns from cultural, historical and economic networks. Results of this comparison are presented in Figure 8. They show that the community structure of the three-layer mobility network is consistently more similar to the community structure of cultural, historical and economic networks than community structure of each layer considered separately. This can serve as a good initial quantitative validation of our hypothesis: when considering different aspects of mobility together in a form of the multi-layer mobility network, one can indeed reveal some patterns more consistent with other observations than patterns discovered by considering any of mobility layers alone.

**Figure 9.**
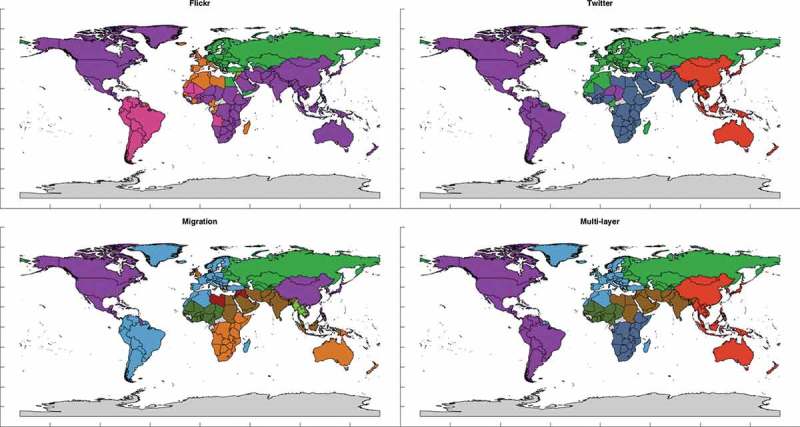
Communities for resolution parameter value equal to 1.0.

**Figure 10.**
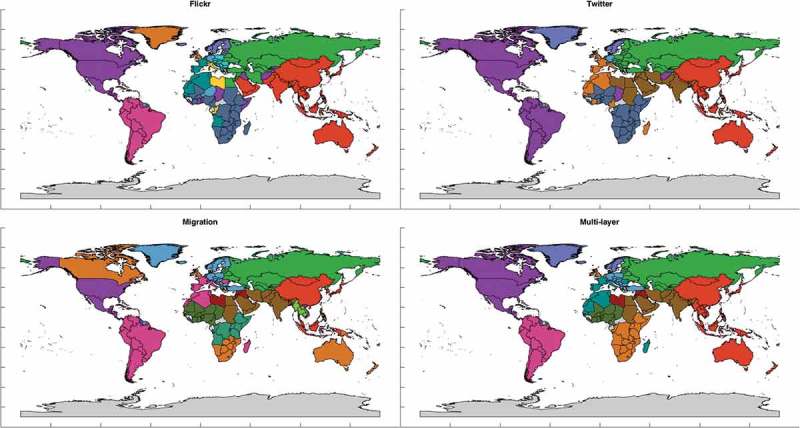
Communities for resolution parameter value equal to 1.5.

**Figure 11.**
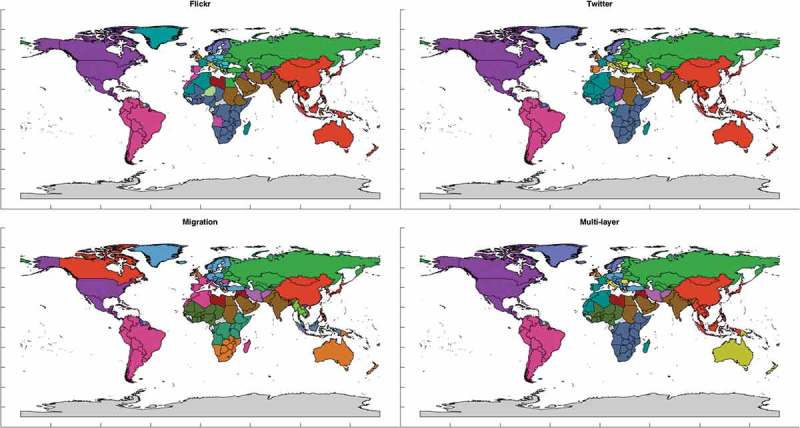
Communities for resolution parameter value equal to 2.0.

In Figures 9–11, we present the results of partitioning with resolution parameter values 1.0, 1.5 and 2.0, respectively, as this is the range where it is possible to visually recognize and makes sense to analyze different communities on a map. Namely, when applying modularity maximization with the default resolution parameter of 1.0 to each layer separately, it leaves us with only 4 and 5 communities for Twitter and Flickr, respectively, while for resolution parameter equal to 2.0, number of detected communities goes up to 17 making it already harder to visually identify and interpret different colors.

From all the figures, we can see that partitions of multi-layer network seem to have less anomalies and are easier to explain than partitions of any layer alone. For example, in the Flickr and migration layers China often appears to be united with North America or even with Canada alone, which is an interesting pattern to analyze and explain. However, the multi-layer network partition based on all the mobility patterns related to the country leaves it as a part of community of Asian countries, which agrees with its overall geopolitical context. In partitions of multi-layer network, one can always clearly see communities of both Americas (for 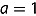 they united into one community), communities of former USSR countries and Arab countries. In case of 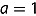 entire Europe is nicely united into one community. And while particular patterns revealed by specific datasets are important to consider as they might point out interesting and sometimes unexpected links, when it comes to an overall regional delineation, the whole variety of such links should be considered. Partitions start to be more complicated for higher values of *a*, but this is because more local patterns are discovered. Nevertheless, in multi-layer network Australia is never united with the United Kingdom, Southeast Asia is always united with the rest of East Asia, south and central Africa form their own communities and China forms community around itself and not united with North American countries, i.e. communities are much more geographically cohesive. Moreover, another interesting property of the mobility networks consistent with the previous findings of Hawelka *et al*. (2014) and Fagiolo and Mastrorillo (2013) is that their partitions are spatially connected even though it is never implied by the method of partitioning. And this property is particularly clearly observed for the multi-layer network partitions.

We do acknowledge that some patterns in community structure of different layers may make sense and could be explained by the processes going on in the world, as different aspects of mobility provide different views. Moreover, patterns observed in long-term movements could differ from those in short-term. Nevertheless, in this research we emphasize that if one wants to study global mobility links between the countries in all their variety (as there are at least two if not more substantially different mobility types), she/he should look at various sources of data (and there are increasingly many such sources being provided by digital media). For example, studying migration patterns might not require looking into business trips as well as delineating touristic regions could be done without taking into account migrations, but for the purpose of inferring geopolitical regions it could be important to consider all the aspects of mobility. Considering just one source that can have its own biases might not be able to uncover general patterns of such a complex system. Alternatively, incorporating various sources can help to account for different factors in reconstructing a general picture of human mobility.

## 6. Conclusions

In this study, we explored records of different types of human mobility: long-term and short-term. We analyzed their similarities and differences and showed that country attractiveness, which is represented with the number of foreign people visiting it, in all three networks follows a log-normal distribution. Moreover, our results showed that normalized weights of links also follow the same distribution with almost the same scaling parameters for Flickr and Twitter networks and much more diverse for the migration one. The existence of a small number of stronger migration flows covering majority of the entire migration activity denotes that people tend to move to rather few major countries of interest, while from touristic or other short-term perspective the choices of destinations is much broader.

We ranked all countries according to their attractiveness for long-term and short-term visits and specifically investigated outlying countries that are highly ranked from one perspective and not from the other. These countries fall into two easily distinguished groups: the first one mostly consists of Arab countries that could be seen as very attractive for immigrants (wealthy, oil-rich countries with a demand for foreign labor), but not that much for tourists; and the second one is composed of developing countries with diverse and exotic nature and quite high population density which attract a lot of tourists, but fewer migrants. Nevertheless, there is also a group of highly developed countries that attract both tourists and immigrants.

We also looked at how different types of mobility could be predicted by existing models. After fitting the gravity model to all layers of our multi-layer mobility network, we showed that long-term mobility is much more constrained by distance than short-term one. This means that when people choose where to live they take into an account distance much more often than when choosing where to go for a trip.

Finally, as we found that different datasets provide different perspectives on human mobility, we combined them into one three-layer mobility network. We showed that considering all three mobility networks together as one single multi-layer network helps us to better describe the structure of the global human society in a way which is more consistent with other types of known links between the countries. Namely, we applied a community detection method to the multi-layer network and to each layer separately and compared all four resulting partitions with those obtained from networks of other existing international connections (i.e. language similarity, colonial relations and international trade). The results showed that the three-layer mobility network provides an underlying structure consistently more similar to the structures behind those international connection networks, compared to each layer taken separately. We also discuss the specific spatial patterns revealed. Therefore, our general conclusion is that considering human mobility network from a multi-layer perspective is crucial as multi-layer mobility networks can reveal some important patterns which single networks cannot.

## Supplementary Material

ijgis-2016-0014-File020.pdf

## Note

1. The Yahoo Webscope Program: http://webscope.sandbox.yahoo.com.

## References

[CIT0001] Abel G.J. and Sander N., 2014 Quantifying global international migration flows. *Science*, 343 (6178), 1520–1522. doi:10.1126/science.1248676 24675962

[CIT0002] Amini A., *et al* 2014 The impact of social segregation on human mobility in developing and industrialized regions. *EPJ Data Science*, 3 (1), 1–20. doi:10.1140/epjds31

[CIT0003] Ana L. and Jain A.K., 2003 Robust data clustering. *In*: *Proceedings of the IEEE Computer Society Conference on Computer Vision and Pattern Recognition*, Vol. 2 IEEE, II–128.

[CIT0004] Arenas A., Fernández V., and Gómez S., 2008 Analysis of the structure of complex networks at different resolution levels. *New Journal of Physics*, 10 (5), 053039. doi:10.1088/1367-2630/10/5/053039

[CIT0005] Bagchi M. and White P., 2005 The potential of public transport smart card data. *Transport Policy*, 12 (5), 464–474. doi:10.1016/j.tranpol.2005.06.008

[CIT0006] Barthélemy M., 2011 Spatial networks. *Physics Reports*, 499 (1), 1–101. doi:10.1016/j.physrep.2010.11.002

[CIT0007] Bojic I., *et al*, 2015a Choosing the right home location definition method for the given dataset. *In*: Liu T.Y., Scollon C.N., and Zhu W., eds. *Social Informatics: 7th International Conference, SocInfo* 2015, Beijing, China, 129-12, 2015, *Proceedings* Cham: Springer International Publishing, 194–208.

[CIT0008] Bojic I., *et al*, 2015b Sublinear scaling of country attractiveness observed from Flickr dataset. *In*: *Proceedings of the IEEE International WIE Conference on Electrical and Computer Engineering* IEEE, 305–308.

[CIT0009] Bojic I., *et al*, 2016 Scaling of foreign attractiveness for countries and states. *Applied Geography*, 73, 47–52. doi:10.1016/j.apgeog.2016.06.006

[CIT0010] Calabrese F. and Ratti C., 2006 Real time Rome. *Networks and Communication Studies*, 20 (3–4), 247–258.

[CIT0011] Danon L., *et al* 2005 Comparing community structure identification. *Journal of Statistical Mechanics: Theory and Experiment*, 2005 (09), P09008. doi:10.1088/1742-5468/2005/09/P09008

[CIT0012] Fagiolo G. and Mastrorillo M., 2013 International migration network: topology and modeling. *Physical Review E*, 88 (1), 012812. doi:10.1103/PhysRevE.88.012812 23944523

[CIT0013] Fagiolo G. and Mastrorillo M., 2014 Does human migration affect international trade? A complex-network perspective. *Plos One*, 9 (5), e97331. doi:10.1371/journal.pone.0097331 24828376 PMC4020834

[CIT0014] Fortunato S. and Barthélémy M., 2007 Resolution limit in community detection. *Proceedings of the National Academy of Sciences*, 104 (1), 36–41. doi:10.1073/pnas.0605965104 PMC1765466 17190818

[CIT0015] Frank M.R., *et al* 2013 Happiness and the patterns of life: a study of geolocated tweets. *Scientific Reports*, 3, 1–9. 10.1038/srep02625 PMC6505625 24026340

[CIT0016] Girardin F., *et al* 2008 Digital footprinting: uncovering tourists with user-generated content. *IEEE Pervasive Computing*, 7 (4), 36–43. doi:10.1109/MPRV.2008.71

[CIT0017] González M., Hidalgo C., and Barabási A.L., 2008 Understanding individual human mobility patterns. *Nature*, 453, 779–782. doi:10.1038/nature06958 18528393

[CIT0018] Good B.H., De Montjoye Y.A., and Clauset A., 2010 Performance of modularity maximization in practical contexts. *Physical Review E*, 81, 046106. doi:10.1103/PhysRevE.81.046106 20481785

[CIT0019] Grauwin S., 2015a Towards a comparative science of cities: using mobile traffic records in New York, London, and Hong Kong In: Helbich M., *et al* eds. *Computational approaches for urban environments*. Cham: Springer International Publishing, 363–387.

[CIT0020] Grauwin S., *et al* 2015b Identifying the structural discontinuities of human interactions. *Arxiv Preprint Arxiv*, 1509, 03149. 10.1038/srep46677 PMC5405407 28443647

[CIT0021] Greenwood M.J., 1985 Human migration: theory, models, and empirical studies. *Journal of Regional Science*, 25 (4), 521–544. doi:10.1111/jors.1985.25.issue-4 12313990

[CIT0022] Hawelka B., *et al* 2014 Geo-located Twitter as proxy for global mobility pattern. *Cartography and Geographic Information Science*, 41 (3), 260–271. doi:10.1080/15230406.2014.890072 27019645 PMC4786829

[CIT0023] Hensel P.R., 2009 *ICOW colonial history data set, version 0.4* [online].: University of North Texas. Available from: http://www.paulhensel.org/icowcol.html [Accessed 271 2017].

[CIT0024] Hoteit S., *et al*, 2014 Estimating human trajectories and hotspots through mobile phone data. *Computer Networks*, 64, 296–307. doi:10.1016/j.comnet.2014.02.011

[CIT0025] Infoplease, 2015 *Languages spoken in each country of the world* [online].: Infoplease. Available from: http://www.infoplease.com/ipa/A0855611.html [Accessed 271 2017].

[CIT0026] Java A., *et al*, 2007 Why we Twitter: understanding microblogging usage and communities. *In*: *Proceedings of the 9th WebKDD and 1st SNA-KDD 2007 Workshop on Web Mining and Social Network Analysis*, WebKDD/SNA-KDD ’07, San Jose, California New York, NY, USA: ACM, 56–65.

[CIT0027] Kang C., *et al*, 2013 Exploring human movements in Singapore: a comparative analysis based on mobile phone and taxicab usages. *In*: *Proceedings of the 2Nd ACM SIGKDD International Workshop on Urban Computing*, UrbComp ’13, Chicago, Illinois New York, NY, USA: ACM, 1–8.

[CIT0028] Kiss J., 2011 *Mapping the world with Flickr and Twitter* [online].: The Guardian. Available from: https://www.theguardian.com/technology/pda/2011/jul/13/flickr-twitter-maps [Accessed 271 2017].

[CIT0029] Kivelä M., *et al* 2014 Multilayer networks. *Journal of Complex Networks*, 2 (3), 203–271. doi:10.1093/comnet/cnu016

[CIT0030] Kung K., *et al* 2014 Exploring universal patterns in human home/work commuting from mobile phone data. *Plos One*, 9 (6), 1–15. doi:10.1371/journal.pone.0096180 PMC4059629 24933264

[CIT0031] Lathia N., Quercia D., and Crowcroft J., 2012 The hidden image of the city: sensing community well-being from urban mobility. *In*: Kay J., *et al*, eds. *Pervasive Computing: 10th International Conference, Pervasive 2012, Newcastle, UK, June 18-22, 2012. Proceedings* Berlin, Heidelberg: Springer Berlin Heidelberg, 91–98.

[CIT0032] Li L., Goodchild M.F., and Xu B., 2013 Spatial, temporal, and socioeconomic patterns in the use of Twitter and Flickr. *Cartography and Geographic Information Science*, 40 (2), 61–77. doi:10.1080/15230406.2013.777139

[CIT0033] Masucci A.P., *et al* 2013 Gravity versus radiation models: on the importance of scale and heterogeneity in commuting flows. *Physical Review E*, 88 (2), 022812. doi:10.1103/PhysRevE.88.022812 24032888

[CIT0034] Mount B., 2010 *Flickr dataset* [online]. *SFGEO. Available From*, http://sfgeo.org/data/tourist-local Accessed 271 2017.

[CIT0035] Mucha P.J., *et al* 2010 Community structure in time-dependent, multiscale, and multiplex networks. *Science*, 328 (5980), 876–878. doi:10.1126/science.1184819 20466926

[CIT0036] Nations U., 2015 *United Nations, Department of Economic and Social Affairs* (2013). *Trends in International Migrant Stock: Migrants by Destination and Origin (United Nations database, POP/DB/MIG/Stock/Rev.2013)* [online].: United Nations. Available from: http://www.un.org/en/development/desa/population/migration/data/index.shtml [Accessed 271 2017].

[CIT0037] Newman M., 2006 Modularity and community structure in networks. *Proceedings of the National Academy of Sciences*, 103 (23), 8577–8582. doi:10.1073/pnas.0601602103 PMC1482622 16723398

[CIT0038] Newman M. and Girvan M., 2004 Finding and evaluating community structure in networks. *Physical Review E*, 69 (2), 026113. doi:10.1103/PhysRevE.69.026113 14995526

[CIT0039] Paldino S., *et al* 2015 Urban magnetism through the lens of geo-tagged photography. *EPJ Data Science*, 4 (1), 1–17. doi:10.1140/epjds/s13688-015-0043-3

[CIT0040] Paldino S., *et al* 2016 Uncovering urban temporal patterns from geo-tagged photography. *Plos One*, 11 (12), e0165753. doi:10.1371/journal.pone.0165753 27935979 PMC5148589

[CIT0041] Pei T., *et al* 2014 A new insight into land use classification based on aggregated mobile phone data. *International Journal of Geographical Information Science*, 28 (9), 1988–2007. doi:10.1080/13658816.2014.913794

[CIT0042] Quercia D., *et al*, 2010 Recommending social events from mobile phone location data. *In*: *Proceedings of the 10th IEEE International Conference on Data Mining* IEEE, 971–976.

[CIT0043] Ratti C., *et al* 2006 Mobile landscapes: using location data from cell phones for urban analysis. *Environment and Planning B*, 33 (5), 727–748. doi:10.1068/b32047

[CIT0044] Ratti C., *et al* 2010 Redrawing the map of Great Britain from a network of human interactions. *Plos One*, 5 (12), 1–6. doi:10.1371/journal.pone.0014248 PMC2999538 21170390

[CIT0045] Sagarra O., Vicente C.P., and Daz-Guilera A., 2013 Statistical mechanics of multiedge networks. *Physical Review E*, 88 (6), 062806. doi:10.1103/PhysRevE.88.062806 24483510

[CIT0046] Santi P., *et al* 2014 Quantifying the benefits of vehicle pooling with shareability networks. *Proceedings of the National Academy of Sciences*, 111 (37), 13290–13294. doi:10.1073/pnas.1403657111 PMC4169909 25197046

[CIT0047] Sgrignoli P., *et al*, 2015 The relation between global migration and trade networks. *Physica A: Statistical Mechanics and Its Applications*, 417, 245–260. doi:10.1016/j.physa.2014.09.037

[CIT0048] Simini F., *et al* 2012 A universal model for mobility and migration patterns. *Nature*, 484 (7392), 96–100. doi:10.1038/nature10856 22367540

[CIT0049] Sobolevsky S., *et al* 2013 Delineating geographical regions with networks of human interactions in an extensive set of countries. *Plos One*, 8 (12), 1–10. doi:10.1371/journal.pone.0081707 PMC3867326 24367490

[CIT0050] Sobolevsky S., *et al* 2014a General optimization technique for high-quality community detection in complex networks. *Physical Review E*, 90 (1), 012811. doi:10.1103/PhysRevE.90.012811 25122346

[CIT0051] Sobolevsky S., *et al*, 2014b Mining urban performance: scale-independent classification of cities based on individual economic transactions. *In*: *Proceedings of the 2nd ASE International Conference on Big data Science and Computing* ASE, 1–10.

[CIT0052] Sobolevsky S., *et al*, 2014c Money on the move: big data of bank card transactions as the new proxy for human mobility patterns and regional delineation. The case of residents and foreign visitors in Spain. *In*: *Proceedings of the IEEE International Congress on Big Data* IEEE, 136–143.

[CIT0053] Sobolevsky S., *et al*, 2015a Scaling of city attractiveness for foreign visitors through big data of human economical and social media activity. *In*: *Proceedings of the IEEE International Congress on Big Data* IEEE, 600–607.

[CIT0054] Sobolevsky S., *et al*, 2015b Predicting regional economic indices using big data of individual bank card transactions. *In*: *Proceedings of the 6th ASE International Conference on Data Science* ASE, 1–12.

[CIT0055] Sobolevsky S., *et al* 2016 Cities through the prism of peoples spending behavior. *Plos One*, 11 (2), e0146291. doi:10.1371/journal.pone.0146291 26849218 PMC4743849

[CIT0056] Szell M., Grauwin S., and Ratti C., 2014 Contraction of online response to major events. *Plos One*, 9 (2), 1–9. doi:10.1371/journal.pone.0089052 PMC3935844 24586499

[CIT0057] Tang L., Wang X., and Liu H., 2012 Community detection via heterogeneous interaction analysis. *Data Mining and Knowledge Discovery*, 25 (1), 1–33. doi:10.1007/s10618-011-0231-0

[CIT0058] Thomee B., *et al* 2016 YFCC100M: the new data in multimedia research. *Communications of the ACM*, 59 (2), 64–73. doi:10.1145/2812802

[CIT0059] Tranos E., Gheasi M., and Nijkamp P., 2015 International migration: a global complex network. *Environment and Planning B: Planning and Design*, 42 (1), 4–22. doi:10.1068/b39042

[CIT0060] Twitter, 2013 *Twitter Streaming API* [online].: twitter. Available from: https://dev.twitter.com/streaming/public [Accessed 271 2017].

[CIT0061] United Nations, 2014 *International trade statistics database* [online].: United Nations. Available from: http://comtrade.un.org [Accessed 271 2017].

[CIT0062] Wilson A.G., 1967 A statistical theory of spatial distribution models. *Transportation Research*, 1 (3), 253–269. doi:10.1016/0041-1647(67)90035-4

[CIT0063] Zipf G.K., 1946 The P1 P2/D hypothesis: on the intercity movement of persons. *American Sociological Review*, 677–686. doi:10.2307/2087063

