# Supplementary material for: Global multi-layer network of human mobility
Source: Int J Geogr Inf Sci. 2017 Mar 13;31(7):1381–402. doi: 10.1080/13658816.2017.1301455 (PMC5426086; doi:10.1080/13658816.2017.1301455)
Supplement: ijgis-2016-0014-File020.pdf [file tgis_a_1301455_sm0310.pdf]

Global multi-layer network of human mobility: Supplementary Information

1 Long-term and short-term mobility

Data about migrations is downloaded from the United Nations website [1]. It represents official statistics about the number of foreigners living in each country. As those people actually relocated for good to live there, we consider such type of movements as a *long-term* mobility. In a contrast to that, the Flickr and Twitter datasets provide information about all the trips, most of which are short-term. Users of these resources who actually travel (i.e., according to our records, were active in more than 1 county) visit on average 1.92 countries in one year (please refer to Figure 1 below).

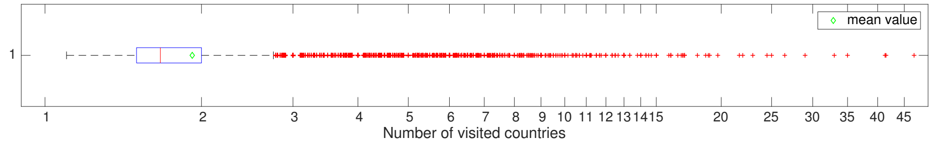

(a)

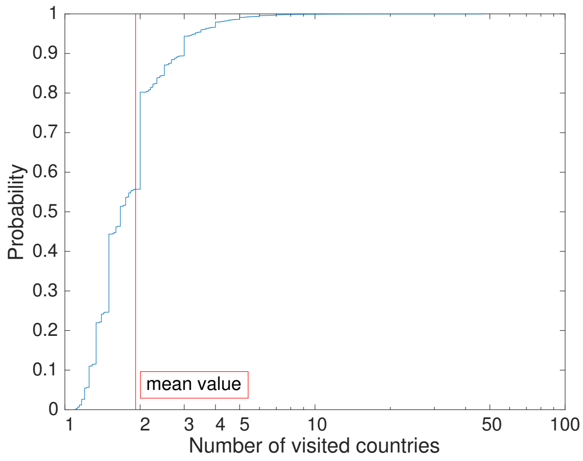

(b)

Fig. 1. Distribution (a) and cumulative distribution (b) of average the numbers of visited countries per one year (axis X is shown on a log-scale).

We also consider time interval between the first and the last days of continuous stay in a country. Obtained results are presented in Figure 2. One can see from it that more than 50% of all 'foreign' activity lasted only for 1 day, about 75% – no more than a week, 85% – no more than a month and 90% – no more than 3 months. These results prove that movements reflected in both Twitter and Flickr datasets represent mainly *short-term* mobility.

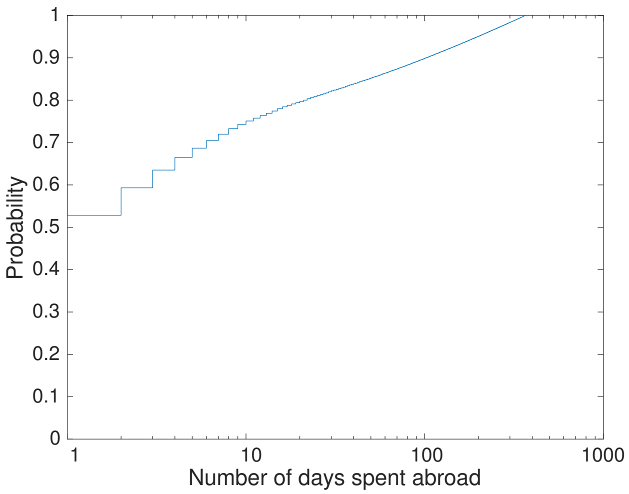

Fig. 2. Cumulative distribution of time interval between cross-country movements (axis X is shown on a log-scale).

2 Countries' performance in attracting visitors

When we compare countries' attractiveness we calculate their rank based on number of foreign residents who visited these countries (we measure rank as inverse of fitted cumulative distribution function). There is a natural tendency for larger countries to attract more visitors. To account for this fact, instead of comparing total attractiveness we can explore how well some countries use their potential to attract visitors compared to other countries. To do so we measure country's 'relative attractiveness' as number of visitors divided by country's population. In the same way as we did for attractiveness, we compare countries' relative attractiveness rank for long-term and short-term visitors. From Figure 3 one can see, that countries that are more attractive for long-term visitors are also the most well performing in doing so. Leading positions from both aspects are taken by rich and rather small countries like Singapore, Luxembourg and Switzerland. Interestingly, most well performing European countries perform

better in attracting short-term visitors, while United States and Australia better attract long-term visitors. That could be partially explained by easiness of traveling between not that big European countries.

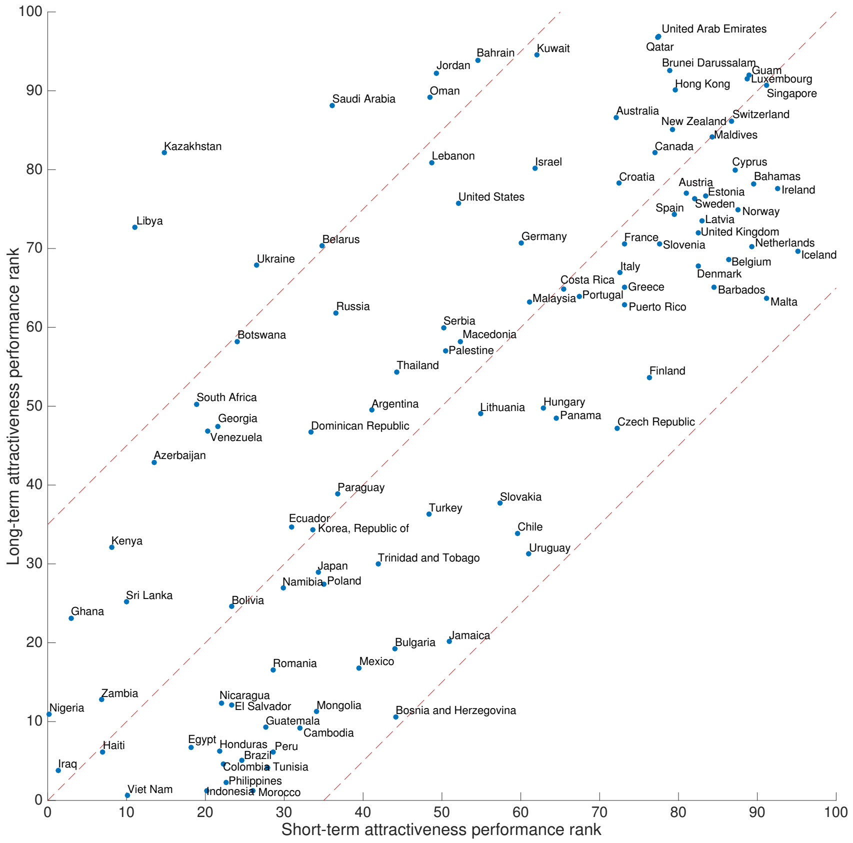

Fig. 3. Comparison of countries' short-term vs. long-term relative attractiveness.

3 Community structure of the supersampled networks

The main claim of our study is that combining data from different sources can lead to qualitatively different results. Putting together three datasets does not just directly mean that we get more data, but it also does mean that we can infer some new knowledge from the information not observed in each of them separately. In order to verify that the community structure does not get affected by simply adding more data to each of the considered datasets we randomly supersampled each network up to the cumulative size of all three of them, i.e., up

to 200 million trips in each network. At first we measured similarity in terms of normalized mutual information (NMI) between community structure of supersampled and original networks. And then we compared community structures of the supersampled and multi-layer network. As one can see from Figure 4, community structure of the supersampled networks always stays almost the same as of the original networks with values of NMI very close to 1.0, while being rather different from the community structure of multi-layer network.

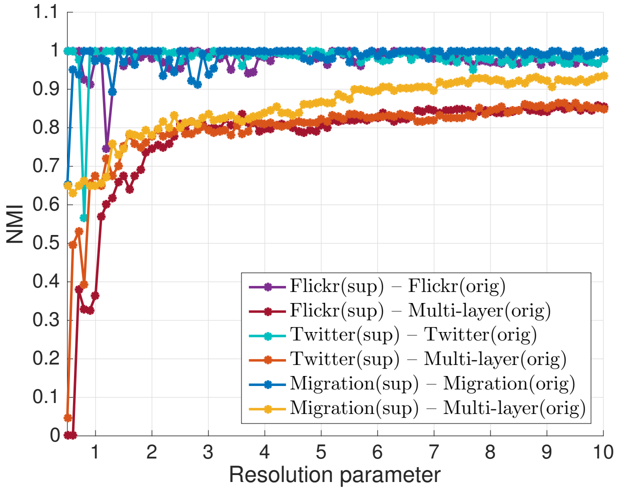

Fig. 4. A contrast in similarity between community structures of supersampled and original against supersampled and multi-layer networks. As it is seen community structure of supersampled network is always very similar to the community structure of original network with NMI values very close to 1, while staying quite different from community structure of multi-layer network.

This experiment shows that simple increase of the data volume indeed does not affect the partitioning of any of the networks considered and stays quite different from the constructed multi-layer network. Based on these findings, we can conclude that the novel partitioning of the multi-layer network does come from a qualitatively new way of combining the network layers, and can not be attributed to a sample size increase alone.

4 Extended range of the resolution parameter values

As we mention in Section 5 of the paper, the number of communities rises up to $60 - 70$ for some of the networks, when the value of the resolution parameter increases to 5.0. At this point most communities consist of 1 to 3 countries facing the geographical granularity of the networks. Because of that further analysis

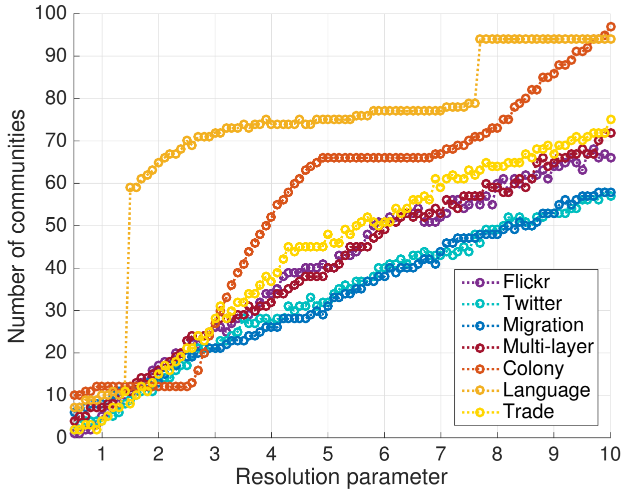

Fig. 5. Number of communities depending on resolution parameter.

of community structure does not make much sense. Here we just want to show that increase of the range of the resolution parameter values up to 10 does not change the fact that average NMI of community structure of multi-layer network is always higher than of any layer taken separately.

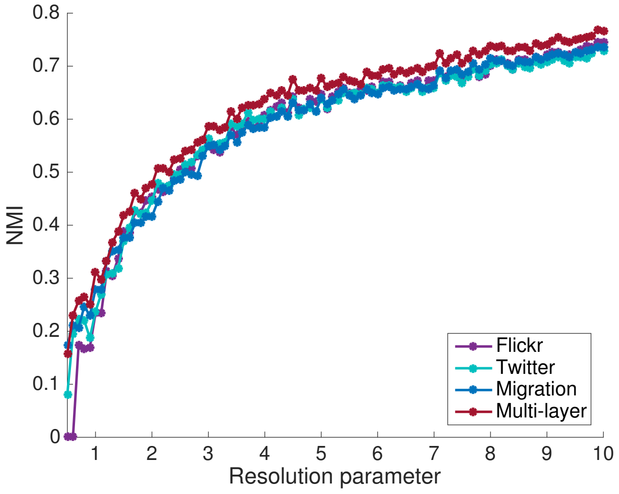

Fig. 6. Similarity of community structure between networks of human mobility and other existing international connections.

Although, at this point we are already facing the network resolution, as number of communities (see Figure 5) rises up to almost 100, meaning that almost all communities consist of 1 or 2 nodes, plot of average NMI shown in Figure 6 indeed does not show any new patterns – the multi-layer network structure is still more consistent with the other existing international connections.

5 Comparison with multi-layer network of non-mobility international connections

As an additional evaluation of the main results, we added comparison of mobility networks to the multi-layer network built of three previously used singular networks (i.e., language, colony and trade networks). We show in Figure 7 that multi-layer mobility network is more similar to multi-layer non-mobility network than any layer of mobility taken alone.

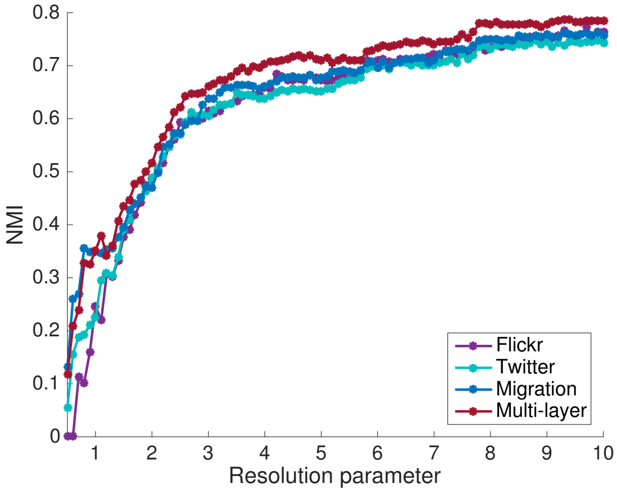

Fig. 7. Similarity of community structure between networks of human mobility and multi-layer network built of colony, language and trade networks.

6 Flickr data year by year

In our analysis we consider a time span of ten years for the Flickr data. This is done in order to overcome sparseness of the Flickr data, so that we can avoid any biases caused by it and make comparison with other denser datasets more fair. However, to make sure that the main results of our analysis are not affected by specific choice of the time frame, we compared results for the entire Flickr

Table 1. Results of gravity model fit to the Flickr data year be year.

Year	R^2 gravity	R^2 l.-n. gravity	α gravity	α l.-n. gravity
2005	0.5514	0.5882	1.0799	1.3479
2006	0.5697	0.6017	1.1213	1.3667
2007	0.5883	0.6216	1.1923	1.4389
2008	0.5968	0.6301	1.2074	1.4556
2009	0.5813	0.6187	1.2377	1.4994
2010	0.5594	0.5979	1.2244	1.4831
2011	0.5247	0.5769	1.1358	1.4748
2012	0.5101	0.5596	1.2020	1.4779
2013	0.4967	0.5640	1.1673	1.5651
2014	0.4024	0.4938	1.2581	1.6772
Mean	0.5381	0.5852	1.1826	1.4787
Std	0.0583	0.0400	0.0561	0.0937

network and for the networks built from the data of each year. First, we fitted gravity model to the data from each year separately and showed that results indeed do not vary much from year to year. In Table 1 we show that obtained values of exponent and r-squared are very similar for all years.

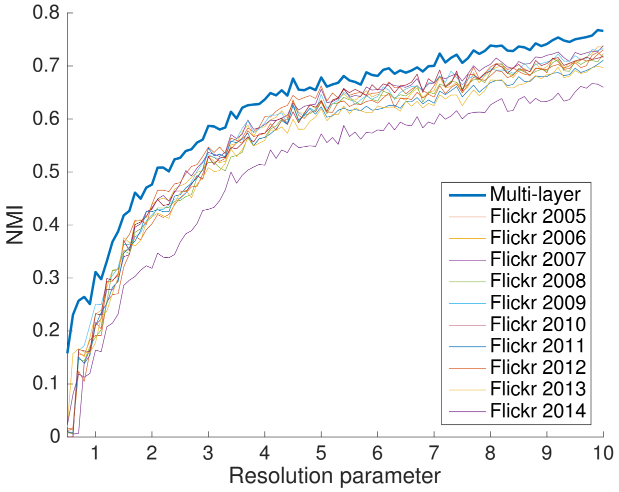

Fig. 8. Similarity of community structure of multi-layer network and networks built from each year of the Flickr data to community structure of the networks of other existing international connections.

Second, we compared community structure of the network of each year to the community structure of the language, colony and trade networks in the same way as we did for three layers of multi-layer network in the main text. In Figure 8 we show that NMI for multi-layer network is still higher than for the network of any year of the Flickr data.

References

1. United Nations: United Nations, Department of Economic and Social Affairs (2013). Trends in International Migrant Stock: Migrants by Destination and Origin (United Nations database, POP/DB/MIG/Stock/Rev.2013) (2015), available from: http://www.un.org/en/development/desa/population/migration/data/index.shtml
